# Efficient virus-mediated genome editing in cotton using the CRISPR/Cas9 system

**DOI:** 10.3389/fpls.2022.1032799

**Published:** 2022-11-16

**Authors:** Jianfeng Lei, Yue Li, Peihong Dai, Chao Liu, Yi Zhao, Yangzi You, Yanying Qu, Quanjia Chen, Xiaodong Liu

**Affiliations:** ^1^ College of Agriculture, Xinjiang Agricultural University, Engineering Research Centre of Cotton, Ministry of Education, Urumqi, China; ^2^ College of Life Sciences, Xinjiang Agricultural University, Urumqi, China

**Keywords:** cotton, Cas9-OE, CLCrV, VIGE, FT-sgRNA, mutation

## Abstract

Plant virus-mediated sgRNA delivery and expression have great advantages; sgRNA expression can rapidly expand and accumulate along with virus replication and movement, resulting in efficient gene editing efficiency. In this study, a VIGE system based on cotton leaf crumple virus (CLCrV) was established using cotton overexpressing Cas9 (Cas9-OE) as the VIGE receptor. CLCrV-mediated VIGE could not only target and knock out the *GhMAPKKK2*, *GhCLA1* and *GhPDS* genes subgroup A and D genome sequences but also achieve double mutation of *GhCLA1* and *GhPDS* genes at the same time. These results verified the effectiveness and efficiency of this system. In addition, the off-target effect assay demonstrated that the CLCrV-mediated VIGE system not only has high gene editing efficiency but also high gene editing specificity in cotton. We further explored whether the *FT-*sgRNA strategy could transport sgRNA to cotton apical meristem (SAM) over long distances to avoid using tissue culture to obtain stable genetic mutants. The results showed that the sgRNA fused with *FT* mRNA at the 5’ end could also efficiently achieve targeted editing of endogenous genes in cotton, but it was difficult to detect heritable mutant progeny. The above results showed that the CLCrV-mediated VIGE system provided an accurate and rapid validation tool for screening effective sgRNAs in cotton.

## Introduction

The acquisition of genetic variant mutants is required for the identification of gene functions and the selection of new varieties of cotton. Random mutations, such as those from artificial mutagenesis and insertional mutagenesis using either T-DNA or transposons, are far from meeting the needs of scientific research and breeding in cotton. The emergence of CRISPR/Cas9 genome editing technology avoids the disadvantage of random mutation blindness and provides important technical tools for realizing precise genetic improvement of cotton. To date, a series of cotton CRISPR/Cas gene editing systems have been established and successively upgraded (Long et al., 2018; Li B et al., 2019; Qin et al., 2020; Wang et al., 2022). However, nearly all genetic variation created through CRISPR/Cas relies on the method of tissue culture, which is time-consuming and laborious for cotton. In addition, some studies have found that sgRNA, one of the key elements of the CRISPR system, is inefficient or invalid at many genomic loci (Behnom and Barbara, 2015). Moreover, it is difficult to predict the activity of sgRNAs by bioinformatics methods (Xie et al., 2014; Zhao et al., 2019). Therefore, before using the CRISPR system to obtain cotton mutants, it is necessary to quickly screen out effective sgRNAs. Protoplast transient transformation (Chen et al., 2017) and *Agrobacterium*-mediated transient transformation of cotton leaves (Gao et al., 2017) are the two most common methods to verify sgRNA activity. However, due to low transient transformation efficiency resulting from the large size of the *Cas9* gene (>4000 bp), gene editing events are difficult to detect, which leads to false negative results.

Virus-induced gene editing (VIGE) is a new technology developed in recent years. Transgenic lines overexpressing Cas9 are used as receptors in the VIGE system; thus, only sgRNAs need to be delivered through viral vectors that contain sgRNA expression elements. More sgRNAs are transcribed, and more target sites are subsequently edited in more cells along with the replication and spread of the virus in plants, which significantly increases the detection rate of gene editing. At present, this strategy has been applied in several model plants and crops: *Arabidopsis* (Ali et al., 2018; Nagalakshmi et al., 2022), *Nicotiana benthamiana* (Ali et al., 2015; Yin et al., 2015; Cody et al., 2017; Ali et al., 2018; Jiang et al., 2019; Ellison et al., 2020; Uranga et al., 2021), wheat (Hu et al., 2019; Li et al., 2021; Chen et al., 2022), soybean (Luo et al., 2021), and maize (Hu et al., 2019). However, application of the VIGE system in cotton has not been reported.

Viruses can efficiently deliver sgRNAs to achieve editing of target genes. However, nearly all current research reports have shown that these editing events can only occur in contemporary plants (Yin et al., 2015; Cody et al., 2017; Jiang et al., 2019). Due to the special mechanism existing in plants themselves, which makes it difficult for viruses to efficiently enter the shoot apical meristem (SAM) (Wu et al., 2020) to achieve gene editing of germ cells, it is difficult to obtain gene-edited progeny. Recently, Ellison reported that mobile RNA elements (*Flowering Locus T*, *FT* and tRNA^Ileu^) were fused to sgRNA and subsequently cloned into RNA viral vectors (tobacco rattle virus, TRV). *FT* and tRNA^Ileu^ could transport sgRNA long distances into the SAM of *Nicotiana benthamiana* and produce heritable gene editing with high efficiency (Ellison et al., 2020). Moreover, it has also been shown that assembly of tRNA^Ileu^ fused to sgRNA in a TRV viral vector can also efficiently obtain heritable gene-edited progeny in *Arabidopsis* (Nagalakshmi et al., 2022). In addition, several studies have reported that unmodified plant virus-delivered sgRNAs can directly achieve heritable gene editing in Cas9-OE *Nicotiana benthamiana* and wheat (Ali et al., 2015; Li et al., 2021; Chen et al., 2022). In previous work, we used the cotton leaf crimp virus (CLCrV)-mediated VIGE system to obtain low-efficiency heritable gene-edited progeny in *Arabidopsis* with the *FT*-sgRNA strategy (Lei et al., 2021). Whether the *FT*-sgRNA strategy or direct use of unmodified sgRNA can achieve heritable gene editing progeny has not been reported in cotton. In this study, we first established a CLCrV-mediated VIGE system in cotton and verified the accuracy of this system in screening effective sgRNAs. At the same time, an *FT*-sgRNA strategy to achieve heritable gene editing in cotton was explored.

## Materials and methods

### Plant materials and growth conditions

Cas9-OE cotton variety YZ-1 (Zhu et al., 2018) and wild-type seeds were soaked in ddH_2_O water for 3 days and then germinated in the dark at 28°C for 48 h. After germination, the seedlings were transplanted into nutrient soil and grown at 28°C under 12 h light/12 h dark conditions. The virus inoculation transformation experiment was performed when the two cotyledons of the cotton seedlings were fully expanded.

### qRT−PCR analysis of Cas9 expression

RNA was isolated from wild-type and Cas9-OE cotton cotyledons, and cDNA was synthesized by reverse transcription. The 188 bp *Cas9* gene fragment was amplified by qRT−PCR, and the *GhUBQ7* gene was used as the reference gene. The primer design is shown in **Supplementary Table 1**. Three technical replicates were set up for each sample. After the reaction, according to the Ct value of the *Cas9* gene and the reference gene, the relative expression levels of the *Cas9* gene in different plants were calculated using the 2^-△△Ct^ method (Livak and Schmittgen, 2001).

### Vector construction

Construction of sgRNA expression vector: Truncated AtU6-26 (331 bp) (Feng et al., 2013) and AtU6 (79 bp) (Nekrasov et al., 2013) promoters were used to drive sgRNA expression. The 20 bp guide RNA (**Supplementary Table 1**) was synthesized according to the method in the literature (Lei et al., 2021), and sequencing was performed to verify the correctness of the sgRNA containing the target sequence. The AtU6-26::sgRNA fragments with target sequences were recovered by *Spe*I and *Pac*I digestion and assembled on CLCrV-A. In addition, an intact editing vector (AtU6-26::*GhMAPKKK2*-sgRNA-Cas9-p1300) targeting the knockout *GhMAPKKK2* gene was constructed to transform wild-type cotton.

Construction of multiple gene editing vector: The *GhCLA1* and *GhPDS* genes were used as targets, and two different AtU6-26 (331 bp) and AtU6 (79 bp) promoters were used to drive sgRNA expression to minimize the probability of gene silencing caused by using the same promoter. The CLCrV-AtU6::*GhPDS*-sgRNA-AtU6-26::*GhCLA1*-sgRNA1 vector was constructed by enzyme digestion and ligation.

Construction of the *FT*-sgRNA expression vector: The AtU6-26::*GhCLA1*-sgRNA1 and AtU6-26::*GhPDS*-sgRNA plasmids was used as the acceptor template, and the *Arabidopsis FT* gene (528 bp) was used as the donor plasmid template. Then, 102 bp *FT* was fused to the 5’ ends of *GhCLA1*-sgRNA1 and *GhPDS*-sgRNA by transfer PCR (Erijman et al., 2014). The primer design is shown in **Supplementary Table 1**. Sequencing was used to verify whether the *FT*-sgRNA vectors were correct.

### Transient transformation of Cas9-OE cotton with CLCrV-sgRNA

For virus inoculation and transient expression, AtU6-26::*GhMAPKKK2*-sgRNA-Cas9-p1300 and different CLCrV-sgRNA expression vectors and CLCrV-B were transformed into *Agrobacterium tumefaciens* strain GV3101. The *Agrobacterium* cultures were inoculated in 15 mL of LB medium (containing 50 μg/mL Rif and 50 μg/mL Kan) at 28°C and 180 rpm and grown overnight at OD600 to approximately 1.6-1.8. The *Agrobacterium* cultures were harvested and resuspended in transformation solution (10 mM MgCl_2_, 10 mM MES and 200 μM acetosyringone), adjusted to an OD600 of approximately 1.0, and incubated at room temperature for 3-4 h in the dark. For virus inoculation, CLCrV-B and derivatives of CLCrV-A were mixed 1:1 in equal proportions. The transformation solution was injected into Cas9-OE cotton leaves with a 1 mL syringe. In addition, AtU6-26::*GhMAPKKK2*-sgRNA-Cas9-p1300 *Agrobacterium* transformants were inoculated into wild-type cotton.

### Mutation detection

To detect whether the target gene was mutated, genomic DNA was extracted from cotton leaves inoculated with AtU6-26::*GhMAPKKK2*-sgRNA-Cas9-p1300 and different CLCrV-A derivatives. A PCR/RE assay (Gao et al., 2017) was used to detect the mutation of the target site, and PCR amplified a genomic fragment containing the target site and appropriate restriction endonuclease site. The amplification primers are shown in **Supplementary Table 1**. The PCR product was digested with restriction enzymes at the target site, while undigested PCR amplicons were cloned into a Blunt Zero cloning vector and sequenced. Finally, the plants with the mutant phenotype were selected, and Hi-TOM high-throughput sequencing was used to detect the gene editing efficiency of the mutant individual plants. The amplification primers are shown in **Supplementary Table 1**.

### Off-target analysis of CLCrV-mediated VIGE systems

Based on the designed *GhCLA1*-sgRNA1 and *GhPDS*-sgRNA sequences, potential off-target sites were identified using CRISPR-GE (Xie et al., 2017) (http://skl.scau.edu.cn/offtarget/) online software. Each potential off-target sequence contained 3-4 bp of mismatched bases, and these potential off-target sequences were used to search the upland cotton database (https://www.cottongen.org/) for the corresponding homologous sequence of each predicted off-target sequence. The primers (**Supplementary Table 1**) were designed to detect the off-target rate, and the fragment covering the off-target sequence was amplified by PCR. The off-target rate was detected by Hi-TOM high-throughput sequencing.

## Results

### Detection of *Cas9* gene expression in cotton Cas9-OE plants

**Figure 1:**
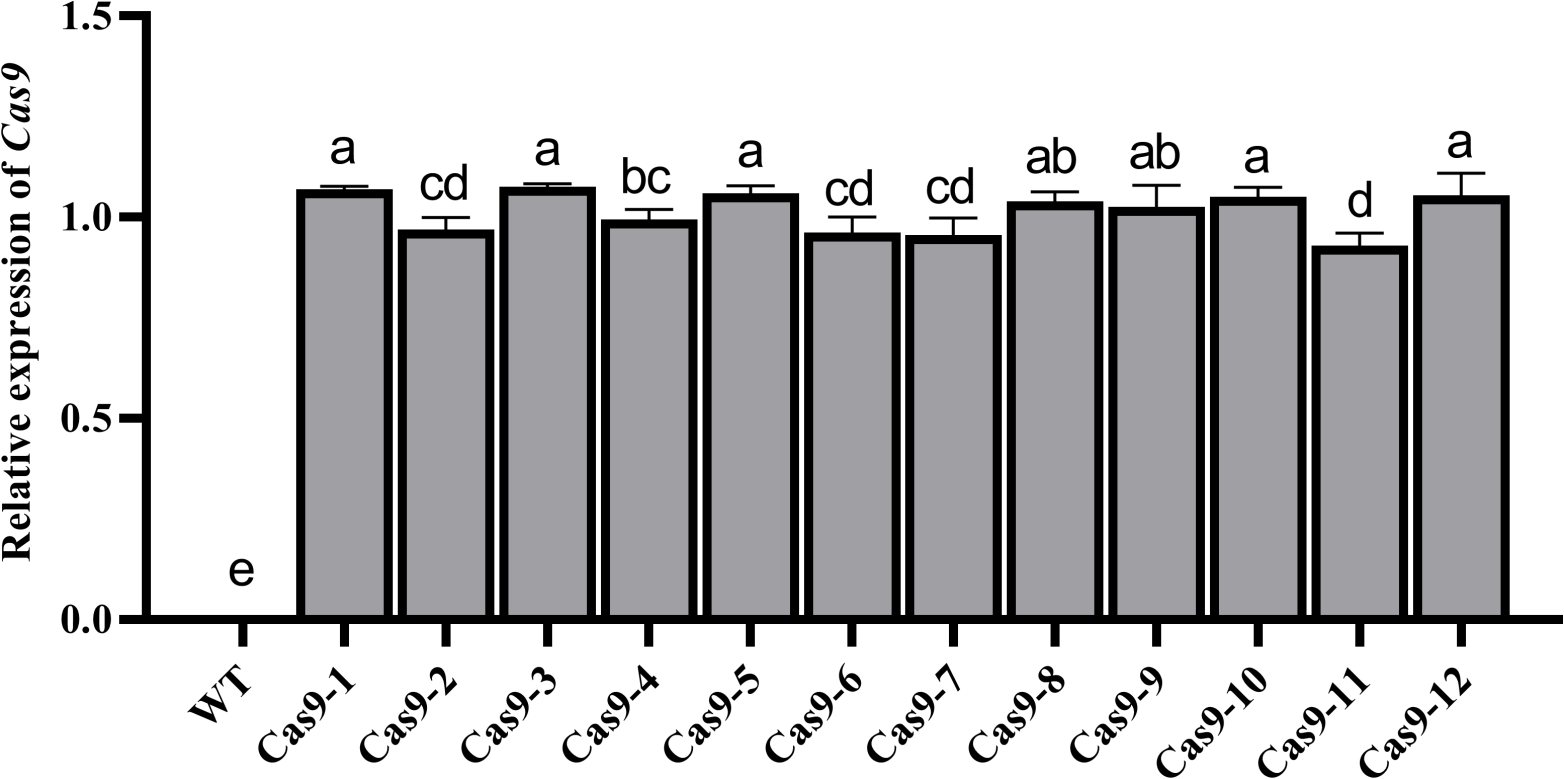
qRT−PCR detection of *Cas9* expression. Different letters indicate significant differences among different treatments at the 0.05 probability level.

In the VIGE system, high expression of *Cas9* is the primary key factor in obtaining gene editing efficiency (Liu et al., 2017; Cho et al., 2018). qRT−PCR analysis showed that the *Cas9* gene was stably expressed in different Cas9-OE cotton plants (Zhu et al., 2018) with only minor differences in expression (**Figure 1**). Cas9-OE transgenic lines with higher *Cas9* expression were selected for further VIGE analysis.

### Development of efficient methods for validating sgRNA activity in cotton

**Figure 2:**
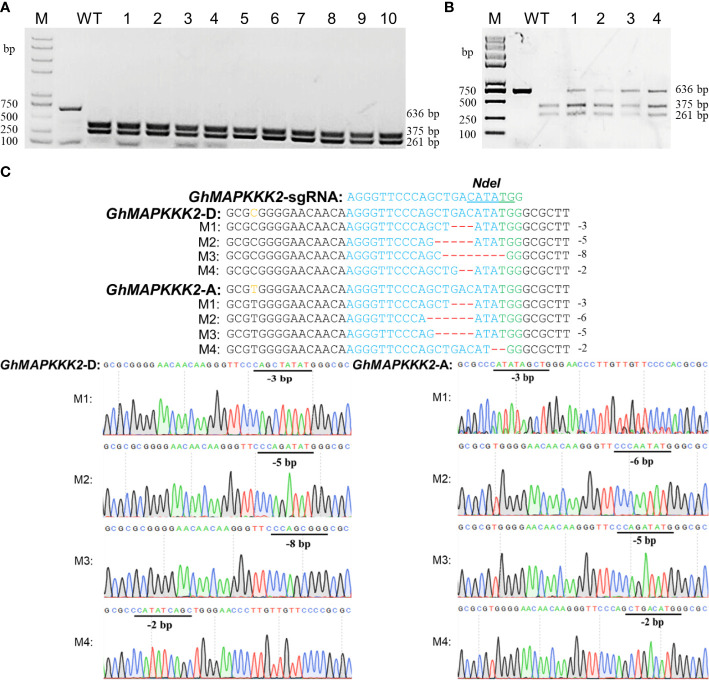
Detection of gene editing effects in two transient transformation modes. **(A)** Detection of AtU6-26::*GhMAPKKK2*-sgRNA-Cas9-p1300 targeted mutations. Wild-type served as a control, and 1-10 were plant numbers. The gel image shows PCR products of the *GhMAPKKK2* gene and digested PCR products with *Nde*I. **(B, C)** Detection of CLCrV-AtU6-26::*GhMAPKKK2*-sgRNA targeted mutations. **(B)** Wild-type served as a control, and 1-4 were plant numbers. The gel image shows PCR products of the *GhMAPKKK2* gene and digested PCR products with *Nde*I. **(C)** The undigested PCR products lacking the *Nde*I site (due to the presence of a mutation) that were subsequently purified, cloned, and analyzed by sequencing. The green color indicates the PAM sequence. The *Nde*I restriction site on the target sequence is underlined in blue. M indicates the mutation sequence. Deletions are shown as red dashes.

CLCrV is a cotton DNA virus whose infection is not affected by coat protein deficiency (Tuttle et al., 2012) and can carry 800 bp of exogenous DNA segments for gene silencing (Gu et al., 2014). In this study, the CLCrV coat protein gene was replaced with a sgRNA expression cassette. To investigate the efficiency of the CLCrV-mediated VIGE system in cotton, the accuracy of two methods to verify sgRNA activity, *Agrobacterium*-mediated transient transformation of intact gene editing vectors with Cas9 and sgRNA (hereafter referred to as ATTI) and CLCrV-mediated transient transformation of the sgRNA expression cassette, were tested. *GhMAPKKK2* (Li et al., 2022) was used as a target, andAtU6-26::*GhMAPKKK2*-sgRNA-Cas9-p1300 and CLCrV-AtU6-26::*GhMAPKKK2*-sgRNA were inoculated into the leaves of wild-type and Cas9-OE cotton plants, respectively. The mutation detection results showed that all the PCR products of the *GhMAPKKK2* gene inoculated with AtU6-26::*GhMAPKKK2*-sgRNA-Cas9-p1300 plants were completely digested, and almost no amplification products remained (**Figure 2A**). In contrast, incomplete digestion of the *GhMAPKKK2* gene PCR product was detected in Cas9-OE plants inoculated with CLCrV-AtU6-26::*GhMAPKKK2*-sgRNA (**Figure 2B**). Further sequencing results showed that different types of base deletion mutations appeared in the *GhMAPKKK2* gene (**Figure 2C**). The results showed that the editing of cotton endogenous genes of subgroups A and D could be achieved efficiently by using the CLCrV delivery sgRNA strategy, which verified the feasibility of sgRNA, but ATTI did not. These results indicated that the CLCrV-mediated VIGE system verified sgRNA activity accurately and avoided false negative results.

**Figure 3:**
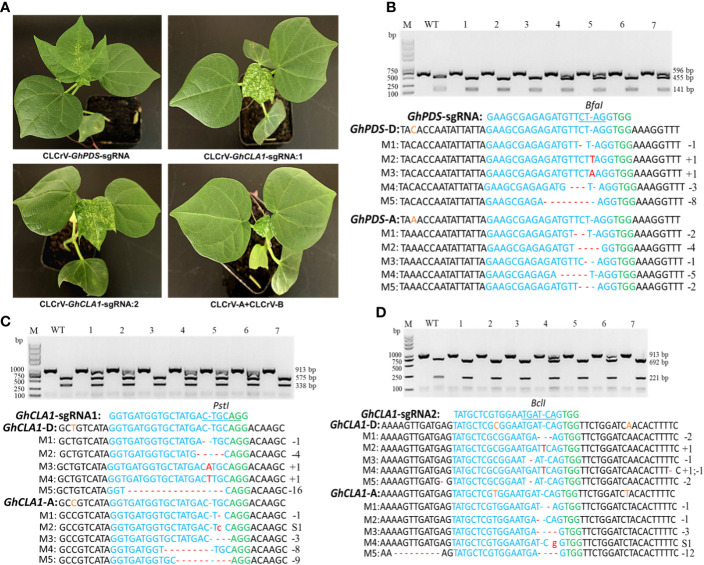
CLCrV-mediated targeted editing of *GhCLA1* and *GhPDS* genes in cotton. **(A)** 20 to 27 days after infiltration, some plant leaves of *GhPDS*-sgRNA, *GhCLA1*-sgRNA1 and *GhCLA1*-sgRNA2 showed a yellow phenotype. Inoculated CLCrV-A and CLCrV-B empty vector cotton served as a control. **(B)** Detection of *GhPDS*-sgRNA targeted mutations. Wild-type served as a control, and 1-7 were plant numbers. The gel image shows PCR products of the *GhPDS* gene and digested PCR products with *Bfa* I, the undigested PCR products lacking the *Bfa* I site (due to the presence of a mutation) that were subsequently purified, cloned, and analyzed by sequencing. **(C)** Detection of *GhCLA1*-sgRNA1 targeted mutations. Wild-type served as a control, and 1-7 were plant numbers. The gel image shows PCR products of the *GhCLA1* gene and digested PCR products with *Pst* I, the undigested PCR products lacking the *Pst* I site (due to the presence of a mutation) that were subsequently purified, cloned, and analyzed by sequencing. **(D)** Detection of *GhCLA1*-sgRNA2 targeted mutations. Wild-type served as a control, and 1-7 were plant numbers. The gel image shows PCR products of the *GhCLA1* gene and digested PCR products with *Bcl* I, the undigested PCR products lacking the *Bcl* I site (due to the presence of a mutation) that were subsequently purified, cloned, and analyzed by sequencing. The green color indicates the PAM sequence. The restriction site on the target sequence is underlined in blue. M indicates the mutation sequence. Insertions are denoted with red capital letters. Deletions are shown as red dashes. Substitutions are denoted with red lowercase letters.

To further test the reliability of the CLCrV-mediated VIGE system for validating sgRNA activity and whether a mutant phenotype can be observed, the *GhCLA1* and *GhPDS* genes, whose mutant leaves were albino, were used as targets. Three target loci were determined (**Supplementary Table 1**), referring to the sequences of the *GhCLA1* and *GhPDS* genes. The leaves of some plants inoculated with *GhPDS*-sgRNA, *GhCLA1*-sgRNA1, and *GhCLA1*-sgRNA2 showed a yellow spot phenotype, while the controls with empty vectors did not (**Figure 3A**). The mutation detection results showed that there were base deletions, insertions, and substitutions in the *GhCLA1* and *GhPDS* genes (**Figures 3B–D** and **Supplementary Figures 1**–**3**). The results indicated that CLCrV-mediated VIGE is a good system for identifying the effectiveness of sgRNA and gene function. In addition, we selected some plants with albino phenotypes to detect the mutation efficiency. The Hi-TOM high-throughput sequencing results showed that the mutation efficiencies of *GhCLA1*-sgRNA1, *GhCLA1*-sgRNA2 and *GhPDS*-sgRNA were 30.01-51.14%, 16.85-42.46%, and 25.74-52.68%, respectively.

### CLCrV-mediated multiple gene editing in cotton

**Figure 4:**
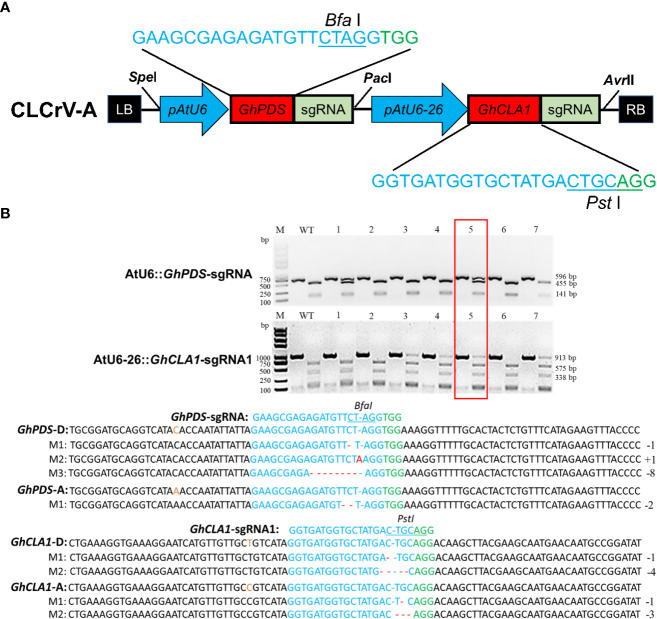
CLCrV-mediated simultaneous editing of *GhCLA1* and *GhPDS* genes in cotton. **(A)** CLCrV-mediated design strategy for simultaneous editing of *GhCLA1* and *GhPDS*. **(B)** Detection of *GhPDS*-sgRNA and *GhCLA1*-sgRNA1 targeted double mutations. Wild-type served as a control, and 1-7 were plant numbers. The gel image shows PCR products of the *GhCLA1* gene and *GhPDS* gene, and digested PCR products with *Pst* I and *Bfa* I. The undigested PCR products lacking the *Pst* I and *Bfa* I site (due to the presence of a mutation) that were subsequently purified, cloned, and analyzed by sequencing. The red box indicates the double mutation of *GhCLA1* and *GhPDS*. The green color indicates the PAM sequence. The restriction site on the target sequence is underlined in blue. M indicates the mutation sequence. Insertions are denoted with red capital letters. Deletions are shown as red dashes.

The CRISPR/Cas9 system can simultaneously cause mutations in multiple genes (Doll et al., 2019; Zeng et al., 2020), which may prove extremely valuable for crops such as cotton, for which transformation is time-consuming and laborious. To verify the multiplex editing capability of the CLCrV-mediated VIGE system in cotton, the *GhCLA1* and *GhPDS* genes described above were used as targets. The DNA segments of AtU6-26::*GhCLA1*-sgRNA1 and AtU6::*GhPDS*-sgRNA were connected in series into the CLCrV-A vector (**Figure 4A**). Mutation detection results showed that a single gene mutation could be detected in most samples. In contrast, double mutations of both *GhCLA1* and *GhPDS* could be detected in only a few samples (**Figure 4B**). Hi-TOM high-throughput sequencing results showed that the double mutation efficiencies of *GhCLA1*-sgRNA1 and *GhPDS*-sgRNA were 8.02% and 25.73%, respectively. The results indicated that the CLCrV-mediated VIGE system could deliver multiple sgRNAs at the same time to enable multigene editing.

### Off-target analysis of CLCrV-mediated VIGE systems

**Figure 5:**
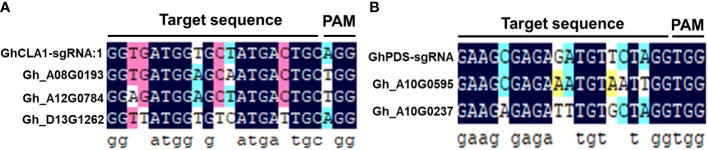
Analysis of 5 potential off-target sites. **(A)** The alignment of 3 predicted off-target sites and the *GhCLA1*-sgRNA1 sequence. **(B)** The alignment of 2 predicted off-target sites and the *GhPDS*-sgRNA sequence.

The occurrence of off-target effects is a key issue in the application of CRISPR/Cas9 gene editing technology in plant functional genomics research and molecular breeding (Li JY, et al., 2019; Wang et al., 2021). To verify whether there was an off-target effect of the CLCrV-mediated VIGE system, five potential off-target sites were retrieved from the cotton database based on *GhCLA1*-sgRNA1 and *GhPDS*-sgRNA (**Figure 5**). The off-target rate was identified in the genomic DNA of leaves inoculated with *GhCLA1*-sgRNA1 and *GhPDS*-sgRNA. The high-throughput sequencing results showed that no off-target phenomenon was found in the five predicted potential off-target sites (**Table 1**), indicating that the CLCrV-mediated VIGE system in cotton not only has high gene editing efficiency but also has high gene editing specificity.

**Table 1:** Detection and analysis of five potential off-target sites.

sgRNA name	Sequence of the putative off-target site	Number of matching bases (include PAM)	Gene name	Region	No. of Examined Events	No. of Off-Target Events
*GhCLA1*-sgRNA1	GGTGATGGAGCAATGACTGCTGG	20	Gh_A08G0193	CDS	985	0	
	GGAGATGGAGCTATGACTGCTGG	20	Gh_A12G0784	CDS	987	0	
	GGTTATGGTGTCATGATTGCAGG	19	Gh_D13G1262	intron	759	0	
*GhPDS*-sgRNA	GAAGCGAGAAATGTAATTGGTGG	19	Gh_A10G0595	CDS	863	0	
	GAAGAGAGATTTGTGCTAGGTGG	19	Gh_A10G0237	intron	963	0	

Red indicates mismatched bases.

### Gene editing in cotton using the *FT*-sgRNA strategy

**Figure 6:**
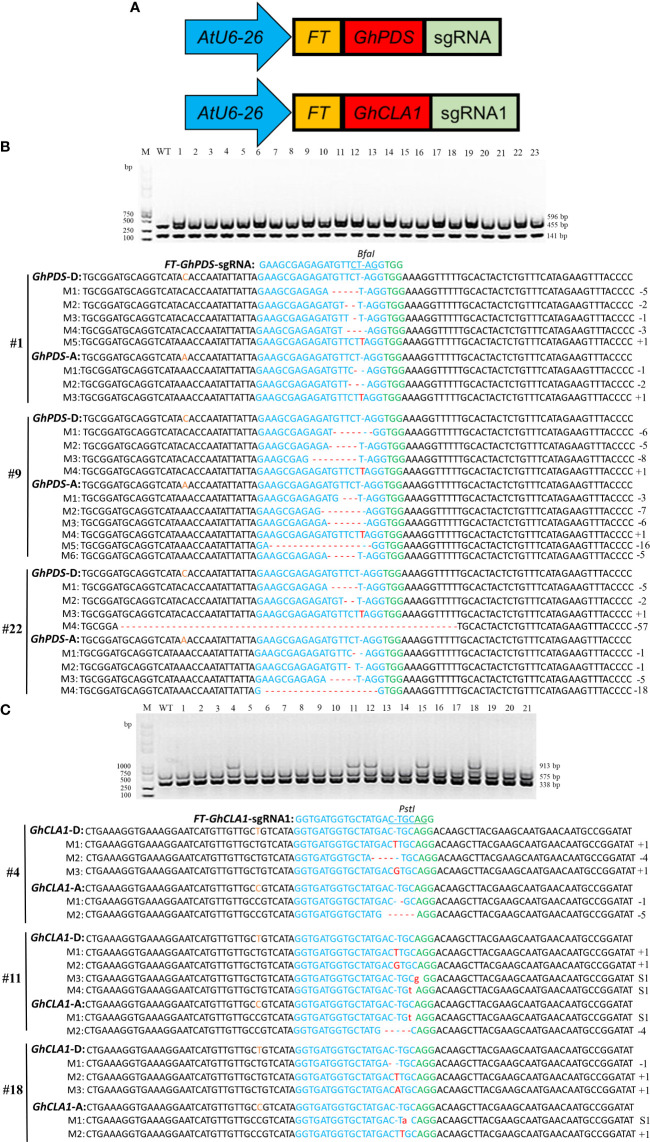
*FT*-sgRNA targeted mutagenesis of *GhPDS* and *GhCLA1* in cotton. **(A)** Truncated *FT* RNA was fused to the 5’ end of *GhPDS*-sgRNA and *GhCLA1*-sgRNA1. **(B)** Detection of *FT*-*GhPDS*-sgRNA targeted mutations. Wild-type served as a control, and 1-23 were plant numbers. The gel image shows the digested PCR products of the *GhPDS* gene with *Bfa* I, and the undigested PCR products lacking the *Bfa* I site (due to the presence of a mutation) were subsequently purified, cloned, and analyzed by sequencing. **(C)** Detection of *FT*-*GhCLA1*-sgRNA1 targeted mutations. Wild-type served as a control, and 1-23 were plant numbers. The gel image shows the digested PCR products of the *GhCLA1* gene with *Pst* I, and the undigested PCR products lacking the *Pst* I site (due to the presence of a mutation) were subsequently purified, cloned, and analyzed by sequencing. Green indicates the PAM sequence. The restriction site on the target sequence is underlined in blue. M indicates the mutation sequence. Insertions are denoted with red capital letters. Deletions are shown as red dashes. Substitutions are denoted with red lowercase letters.

Referring to the *FT*-sgRNA strategy that could achieve heritable gene editing progeny in *Nicotiana benthamiana* and *Arabidopsis* (Ellison et al., 2020; Lei et al., 2021), it was further verified whether this strategy could work in cotton. *GhCLA1* and *GhPDS* were used as targets, and the *FT* (102 bp) gene was fused to the 5’ end of *GhPDS*-sgRNA and *GhCLA1*-sgRNA1, respectively (**Figure 6A**). The two fusion expression vectors were transformed into Cas9-OE cotton leaves. Mutation detection results showed that the PCR products of the *GhPDS* gene from eleven plants transformed with *FT*-*GhPDS*-sgRNA were incompletely digested, and three of them (#1, #9 and #22) were selected for mutation detection. Deletions or insertions were detected in the *GhPDS* gene (**Figure 6B** and **Supplementary Figure 4**). For the plants transformed with *FT*-*GhCLA1*-sgRNA1, PCR products from five plants were incompletely digested, and the genotypes of three plants (#4, #11 and #18) were deletions, insertions and substitutions of bases in the *GhCLA1* gene (**Figure 6C** and **Supplementary Figure 5**). The above results showed that fusion of 102 bp *FT* mRNA to the 5’ end of sgRNA could also effectively achieve gene editing in cotton. High-throughput sequencing results showed that the editing efficiency of *FT*-sgRNA for *GhCLA1* and *GhPDS* genes was 23.98-50.07% and 28.26-55.43%, indicating that there was no significant difference between unmodified sgRNA and *FT*-sgRNA with editing efficiency.

**Figure 7:**
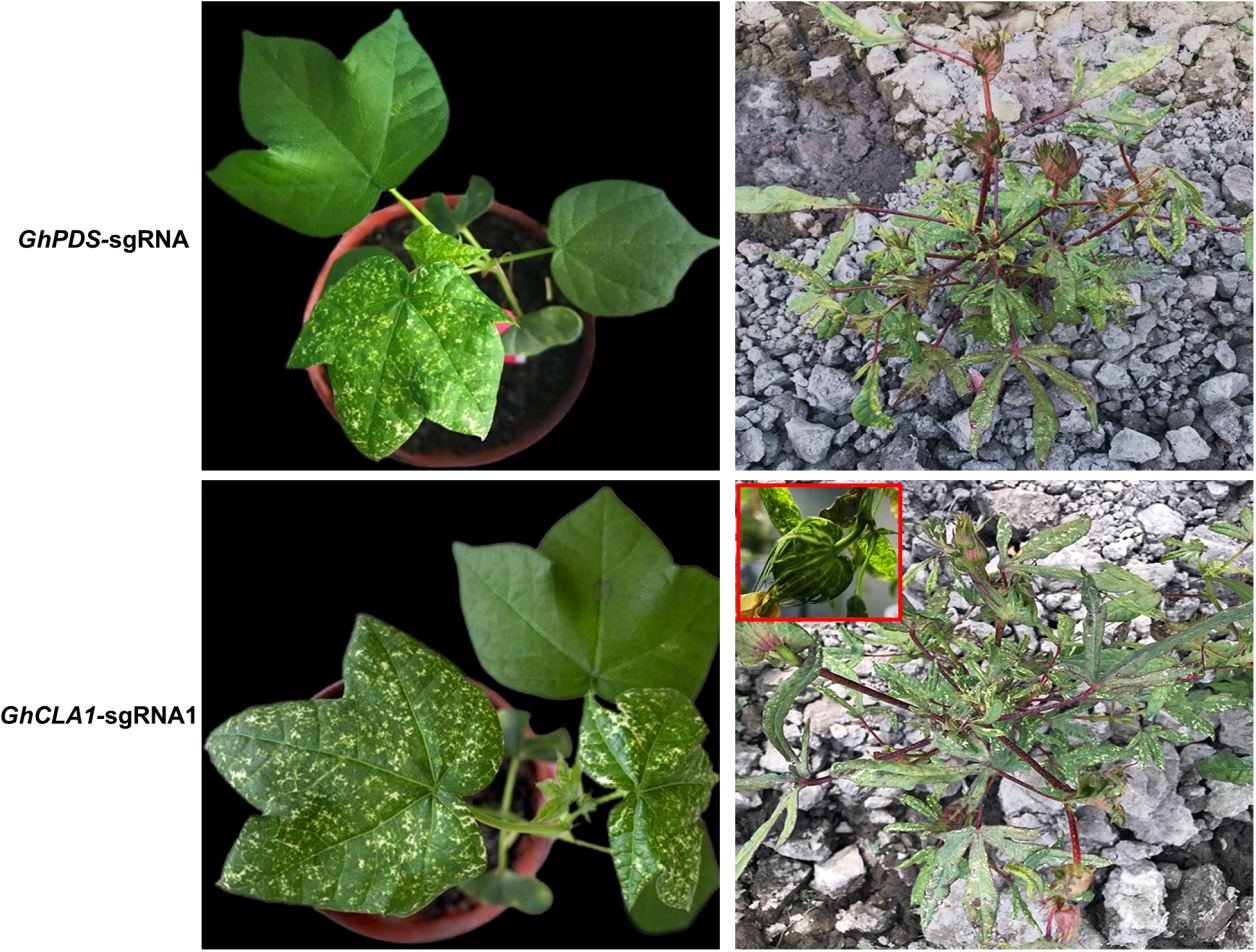
Mutation phenotypes of *GhPDS* and *GhCLA1* genes in cotton. In the field, the incomplete albino phenotype of these two genes persisted into the reproductive growth stage. The red box shows the cotton boll albinism mutant phenotype.

Whether CLCrV-mediated VIGE can achieve gene editing in germ cells of cotton and obtain mutant seeds for target genes remains to be determined. The plants inoculated with unmodified sgRNA and *FT*-sgRNA continued to grow for 50-60 days, and the phenotype of incomplete albinism of plant leaves continued until the reproductive growth stage (**Figure 7**). Seeds were harvested from these plants and sown. The mutant phenotype was observed after 15-20 days. However, no obvious phenotypes were observed in any progeny of plants inoculated with unmodified sgRNA (M1, n=158) or inoculated with *FT*-sgRNA (M1, n=109). The high-throughput sequencing analysis of all M1 generation plants showed that only different base substitution types of *GhPDS* and *GhCLA1* genes in the target sequence were detected, the frequency was less than 3% similar to the control, and no mutation types of base insertion and deletion were detected (**Supplementary Figure 6**). We speculate that these variations may be caused by random mismatches during PCR amplification rather than the result of gene editing.

## Discussion

Whole-genome sequencing of cotton has been completed. However, the function of cotton genes and the biological significance of all DNA elements in the whole genome are still poorly understood. Mutants from target site mutations in cotton are required for the above research, but they are not yet available. The emergence of CRISPR/Cas gene editing technology provides a powerful reverse genetics tool for cotton functional genomics research. Although cotton CRISPR/Cas gene editing systems have been established and a few gene-edited cotton plants have been obtained with the system, it is inconceivable to build gene mutant libraries of cotton and achieve precise genetic improvement at the genome-wide level. Therefore, it is worth establishing a gene editing system with high efficiency and easy operation in cotton.

Plant viral vectors are ideal tools for the delivery and transient expression of exogenous genes in plants and are often used for gene silencing and gene editing in plants. The CRISPR/Cas system is large (usually >5 kb); therefore, it is difficult to use viruses to deliver the system into plant cells by virus infection (Yin et al., 2015; Cody et al., 2017; Ali et al., 2018; Jiang et al., 2019), except for negative-strand RNA sonchus yellow net rhabdovirus (SYNV) (Ma et al., 2020). CLCrV is a DNA virus whose genome has been modified to establish the VIGS system in upland cotton (Gu et al., 2014; Zhang et al., 2016). However, it is still unknown whether CLCrV can also be used as a sgRNA delivery tool for targeted editing of endogenous genes in cotton. Although the CLCrV vector is not suitable for expressing the *Cas9* gene, it is sufficient to express short sgRNAs. Therefore, Cas9-OE cotton plants were used as VIGE receptors, and sgRNA was delivered by CLCrV, which can accumulate and spread rapidly in cotton. For *GhMAPKKK2*-sgRNA, *GhCLA1*-sgRNA and *GhPDS*-sgRNA, mutation analysis results demonstrated that this system could efficiently edit endogenous genes in subgenomes A and D of cotton (**Figures 2**, **3**). Furthermore, double mutation of *GhPDS* and *GhCLA1* genes could be achieved by expressing multiple sgRNAs simultaneously in cotton (**Figure 4**), which provides a favorable tool for studying the functions of gene families and signaling pathways in cotton. For potential off-target sites, no gene editing events were detected. Our experiments demonstrated that the CLCrV-mediated VIGE system not only has high gene editing efficiency but also high gene editing specificity in cotton (**Figure 5** and **Table 1**).

Similar to other plant viruses, such as PEBV (Ali et al., 2018), TMV (Cody et al., 2017), BNYVV (Jiang et al., 2019), BSMV (Hu et al., 2019), and CaLCuV (Yin et al., 2015)-mediated VIGE systems, the CLCrV-mediated VIGE system could efficiently achieve targeted editing of endogenous genes in *Arabidopsis* (Lei et al., 2021) and cotton. However, these editing events can only occur in contemporary plants, and it is difficult to obtain heritable gene-edited offspring. Although the strategy of fusing sgRNA with *FT* (Ellison et al., 2020; Lei et al., 2021) can avoid the use of tissue culture to obtain heritable gene-edited plants, it currently only works in *Nicotiana benthamiana* and *Arabidopsis*, while its application on cotton has not been reported. In this study, we continued to investigate whether the *FT*-sgRNA strategy could generate heritable gene-edited offspring in cotton based on the CLCrV-mediated VIGE system. Similar to the results in *Arabidopsis* (Lei et al., 2021), sgRNAs fused with *FT* mRNA at its 5’ end could also efficiently achieve targeted gene editing in cotton (**Figure 6**). However, mutations in target sites were not detected in M1 progeny seedlings from the parental plants infected with *FT*-sgRNA and unmodified sgRNA. Therefore, we speculate that CLCrV virus or *FT*-sgRNA cannot diffuse into cotton germ cells to achieve heritable editing, or even if it can enter the germ cells, the efficiency of mutation is very low, indicating that CLCrV does not belong to the virus type that can colonize the SAM or *FT* could not transport sgRNA into the SAM of cotton. Recently, it was shown that transgenic *Nicotiana attenuata* with the expression of *Cas9* driven by the germ cell-specific *RPS5A* promoter was used as the VIGE receptor, and TRV-mediated VIGE was used to obtain low-efficiency heritable gene editing in the offspring (Oh and Kim, 2021). Even though the heritability of this approach is low, it provides another strategy for the targeted creation of mutant materials in plants that are difficult to genetically transform without going through a tissue culture process.

The above results showed that the CLCrV-mediated VIGE system can effectively achieve targeted editing of cotton endogenous genes but cannot or at least is difficult to achieve heritable gene editing. BSMV has been found to be a plant virus that can colonize SAM and achieve heritable gene editing (Lin and Langenberg, 1985; Li et al., 2021); it is of interest to determine whether there is a certain type of virus that can colonize SAM for heritable gene editing in cotton. This requires extensive screening of the various types of viruses that can enter the SAM of cotton. If not, other strategies will need to be explored to achieve heritable editing. Our study provides an accurate and rapid validation tool for screening effective sgRNAs in cotton.

## Publisher’s note

All claims expressed in this article are solely those of the authors and do not necessarily represent those of their affiliated organizations or those of the publisher, the editors and the reviewers. Any product that may be evaluated in this article, or claim that may be made by its manufacturer, is not guaranteed or endorsed by the publisher.

## Data availability statement

The data presented in the study are deposited in the JL repository, accession number DOI: 10.3389/fpls.2022.1032799. Email: kyleijianfeng@163.com.

## Author contributions

JL and XL designed the experiments. JL performed most of the experiments and analyzed the data, other authors assisted in experiments. L and XL wrote the manuscript. All authors contributed to the article and approved the submitted version.

## Funding

This work was supported by the Xinjiang Uygur Autonomous Region Major Science and Technology Project (2021A02001-3).

## Acknowledgments

We thank Professor Xueping Zhou from Zhejiang University for providing the CLCrV-A and CLCrV-B vectors. We thank Professor Jie Sun from Shihezi University for providing the Cas9-OE cotton seeds.

## Conflict of interest statement

The authors declare that the research was conducted in the absence of any commercial or financial relationships that could be construed as a potential conflict of interest.

## Publisher’s note

All claims expressed in this article are solely those of the authors and do not necessarily represent those of their affiliated organizations, or those of the publisher, the editors and the reviewers. Any product that may be evaluated in this article, or claim that may be made by its manufacturer, is not guaranteed or endorsed by the publisher.
