# Supplementary material for: Efficient virus-mediated genome editing in cotton using the CRISPR/Cas9 system
Source: Front Plant Sci. 2022 Nov 16;13:1032799. doi: 10.3389/fpls.2022.1032799 (PMC9709312; doi:10.3389/fpls.2022.1032799)
Supplement: Supplementary file 1 [file DataSheet_1.docx]

**Supplementary material**

**Efficient Virus-Mediated Genome Editing in Cotton Using the CRISPR/Cas9 System**

Jianfeng Lei^1^, Yue Li^2^, Peihong Dai^2^, Chao Liu^2^, Yi Zhao^2^, Yangzi You^2^, Yanying Qu^1^, Quanjia Chen^1*^, and Xiaodong Liu^2*^

**^1^** College of Agriculture, Xinjiang Agricultural University, Engineering Research Centre of Cotton, Ministry of Education, Urumqi, China

**^2^** College of Life Sciences, Xinjiang Agricultural University, Urumqi, China

**^*^**Correspondence: Xiaodong Liu ([xiaodongliu75@aliyun.com](mailto:xiaodongliu75@aliyun.com)); Quanjia Chen (chqjia@126.com)

The following Supplementary material is available for this article:

**Fig 1.** DNA sequence of targeted editing of *GhPDS* by *GhPDS*-sgRNA

**Fig 2.** DNA sequence of targeted editing of *GhCLA1* by *GhCLA1*-sgRNA1

**Fig 3.** DNA sequence of targeted editing of *GhCLA1* by *GhCLA1*-sgRNA2

**Fig 4.** DNA sequence of targeted editing of *GhPDS* by *FT*-*GhPDS*-sgRNA

**Fig 5.** DNA sequence of targeted editing of *GhCLA1* by *FT*-*GhCLA1*-sgRNA1

**Fig 6.** DNA sequence of *GhCLA1* and *GhPDS* genes in cotton M1 generation

**Table 1.** Primer sequences involved in this study

**Fig 1.** DNA sequence of targeted editing of *GhPDS* by *GhPDS*-sgRNA.


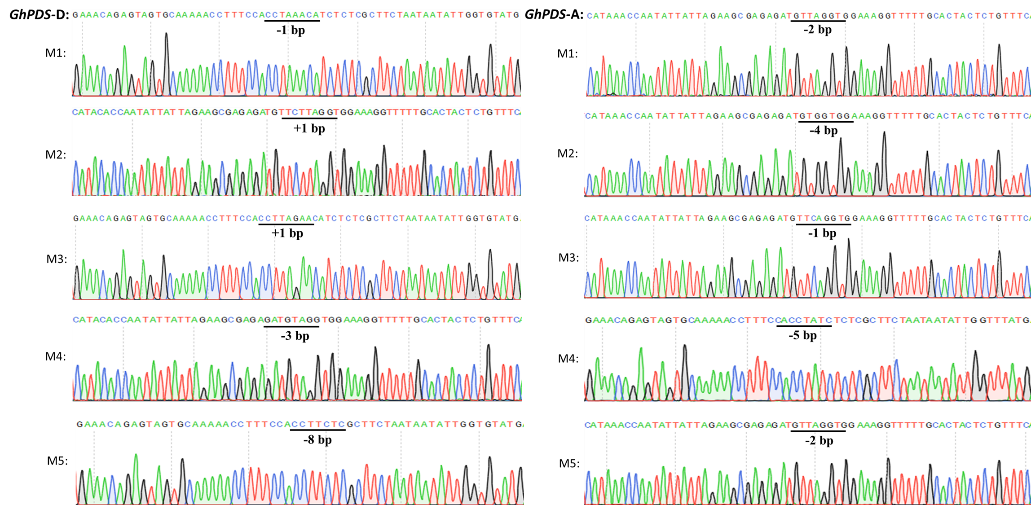


**Fig 2.** DNA sequence of targeted editing of *GhCLA1* by *GhCLA1*-sgRNA1.


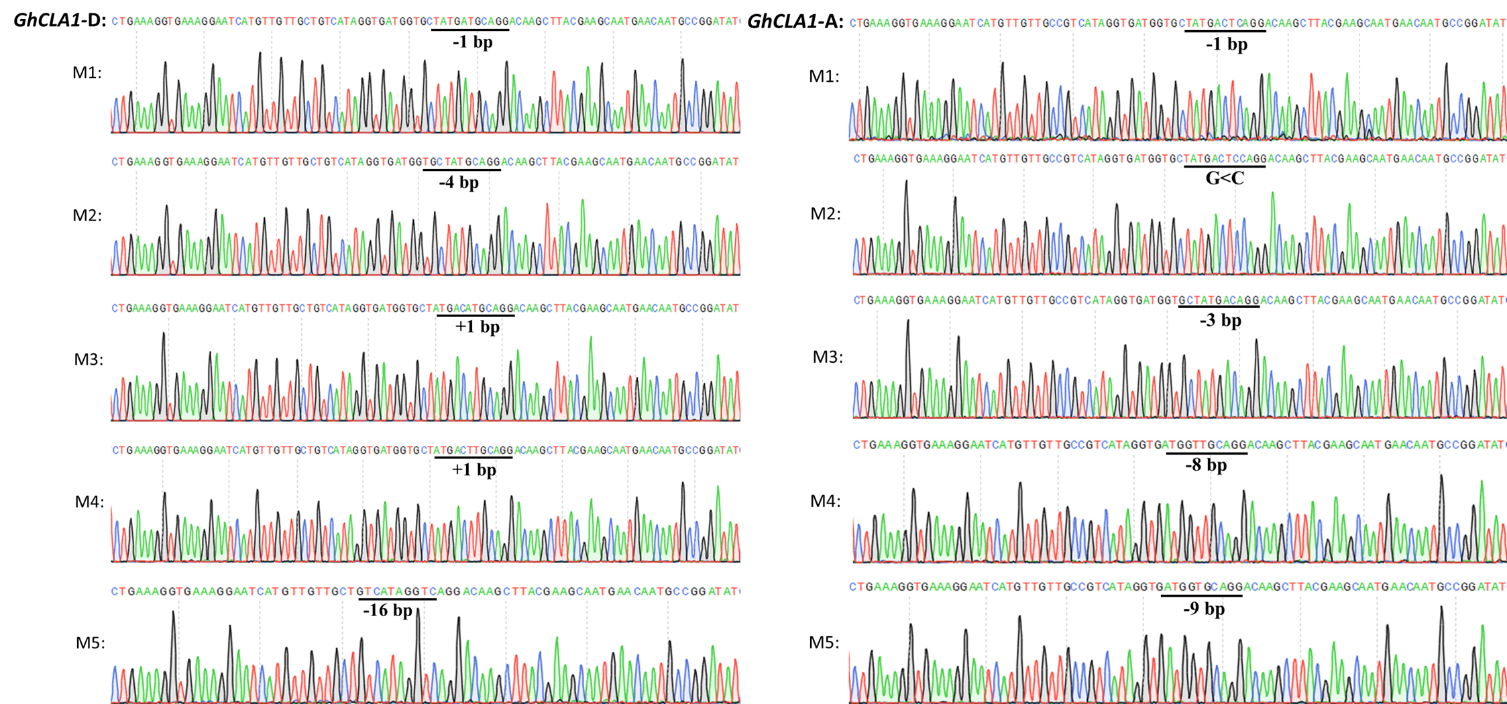


**Fig 3.** DNA sequence of targeted editing of *GhCLA1* by *GhCLA1*-sgRNA2.


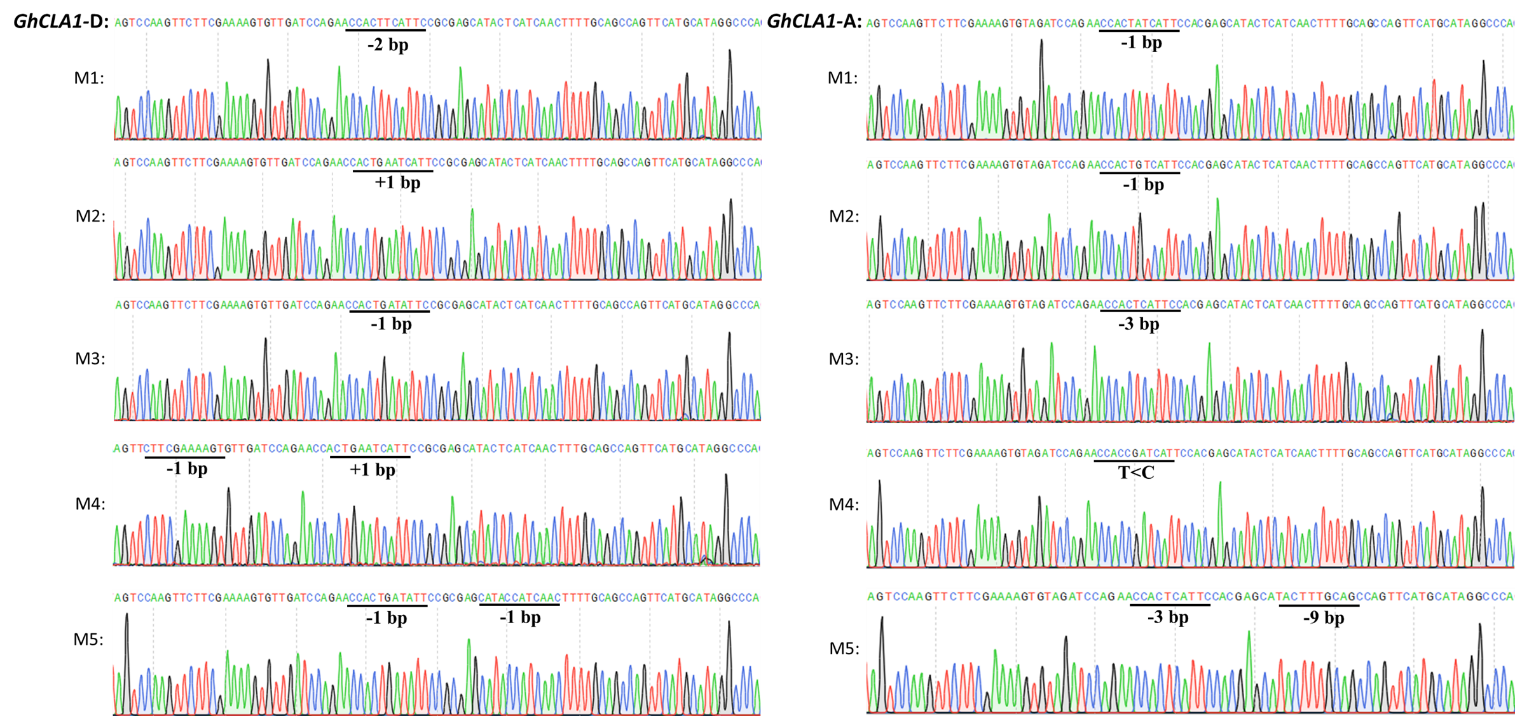


**Fig 4.** DNA sequence of targeted editing of *GhPDS* by *FT*-*GhPDS*-sgRNA.


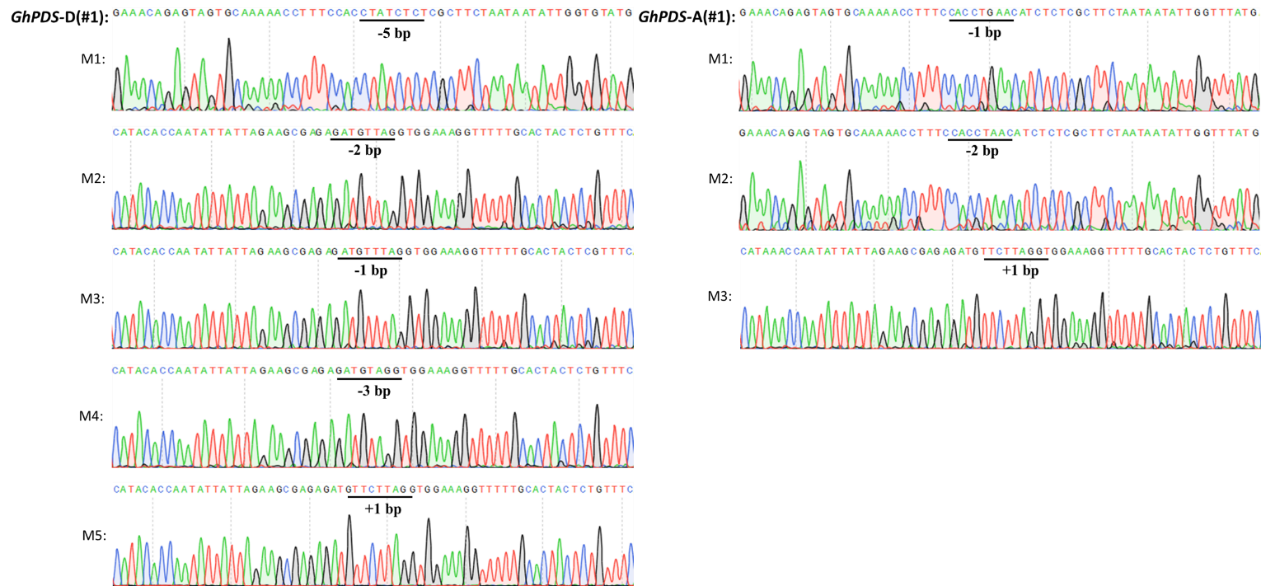

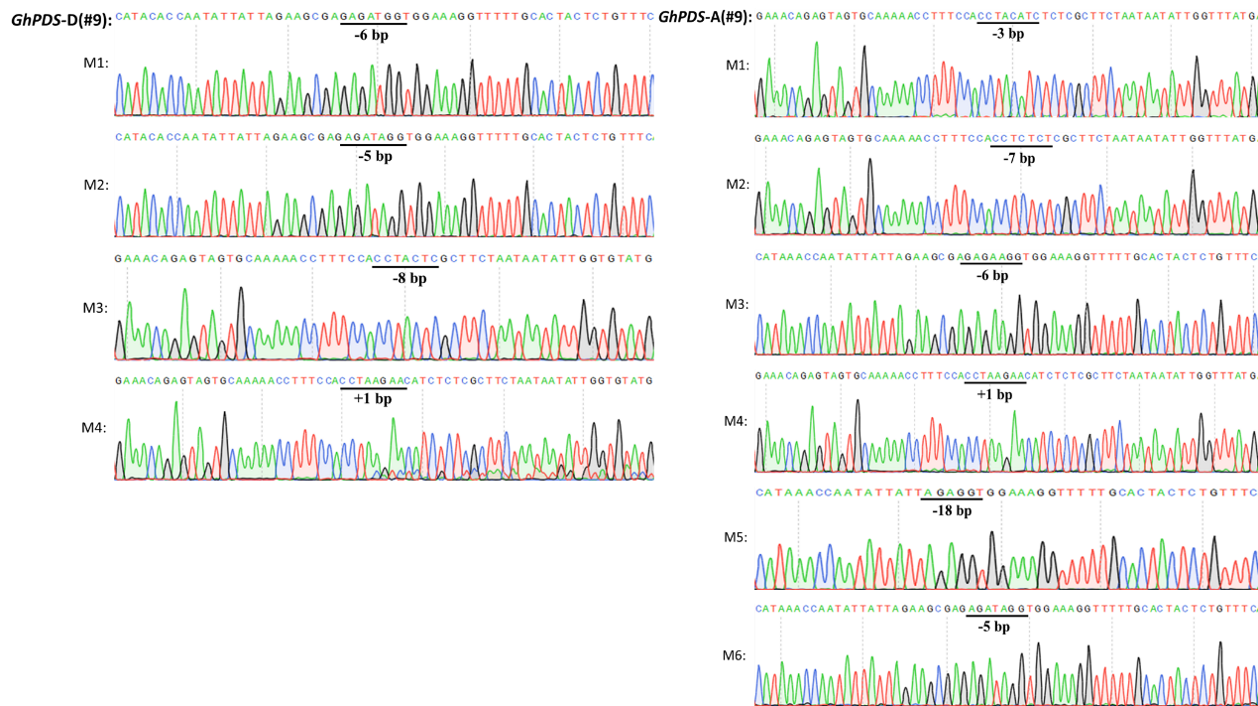

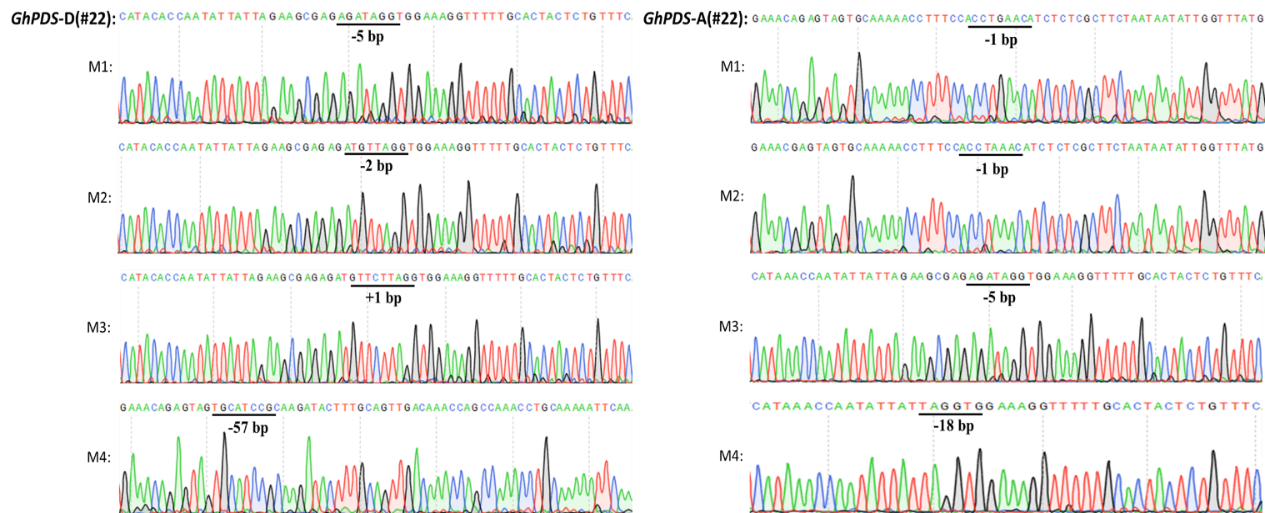


**Fig 5.** DNA sequence of targeted editing of *GhCLA1* by *FT*-*GhCLA1*-sgRNA1


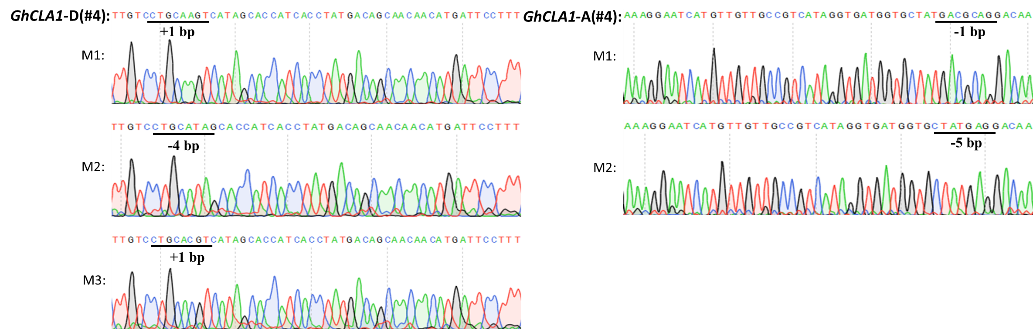


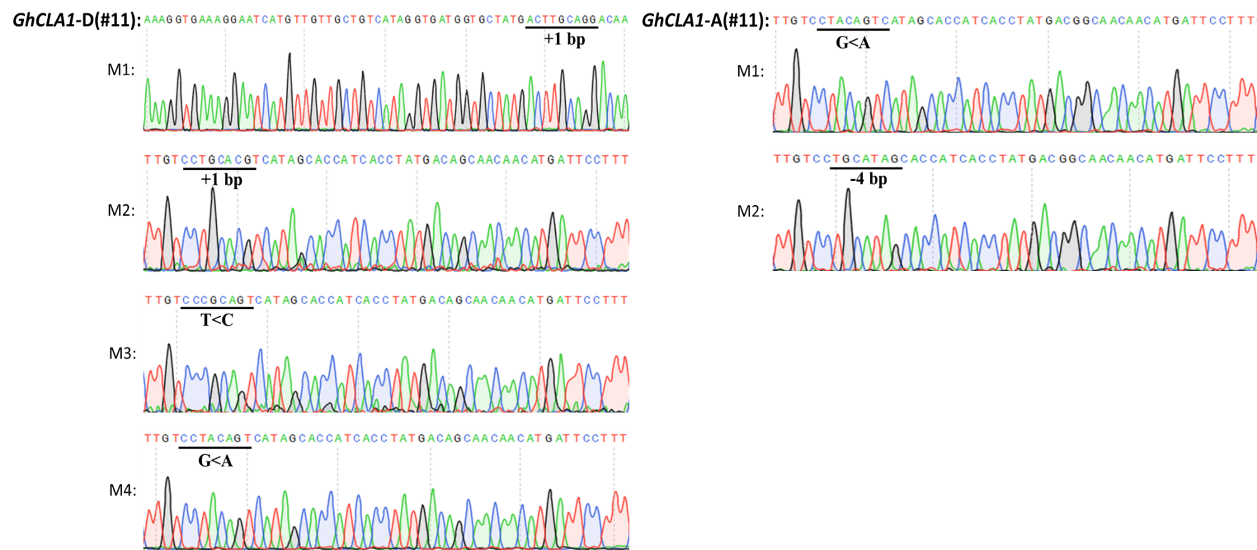


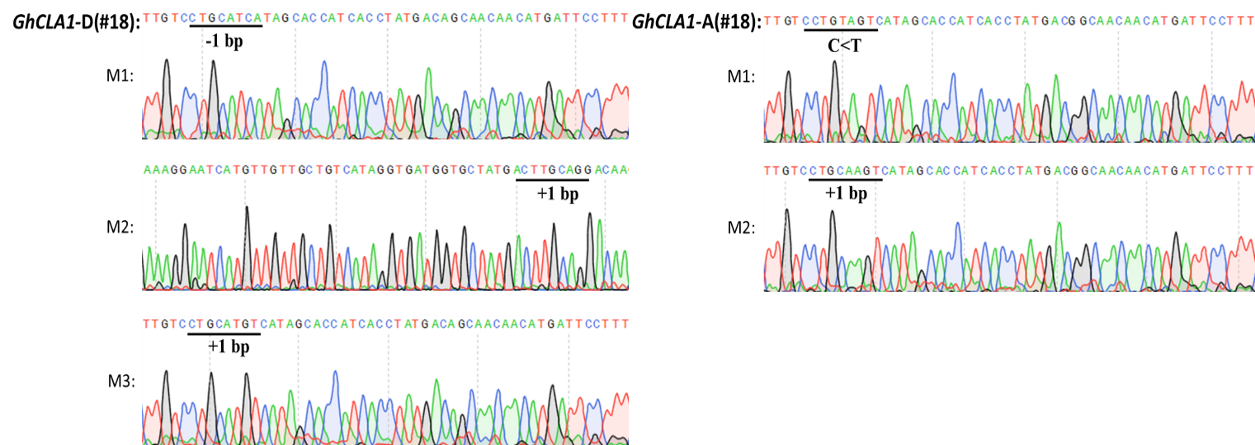


**Fig 6.** DNA sequence of *GhCLA1* and *GhPDS* genes in cotton M1 generation

**
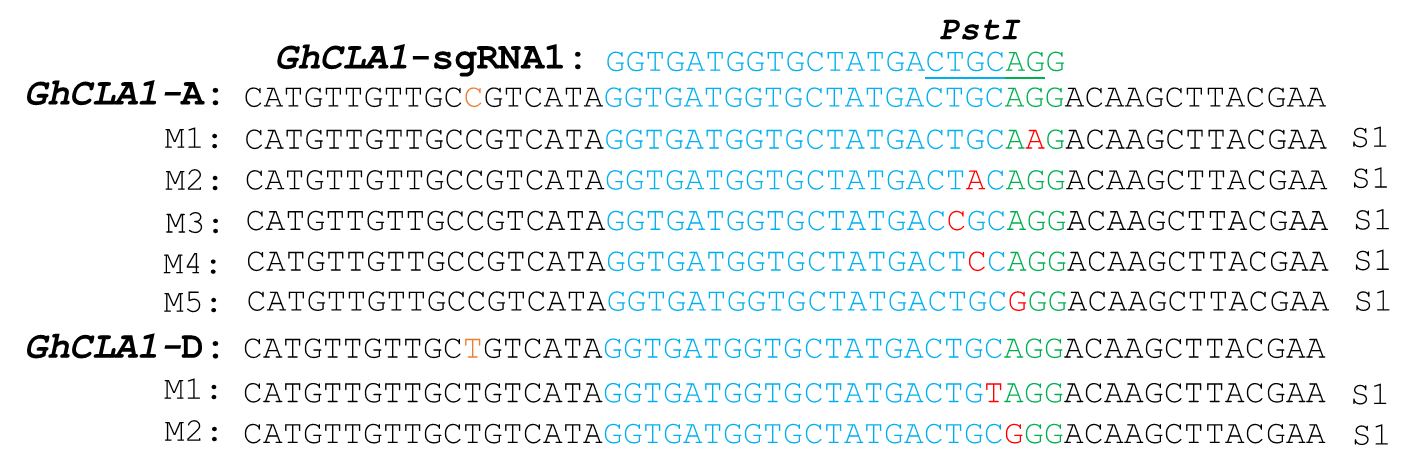
**

**
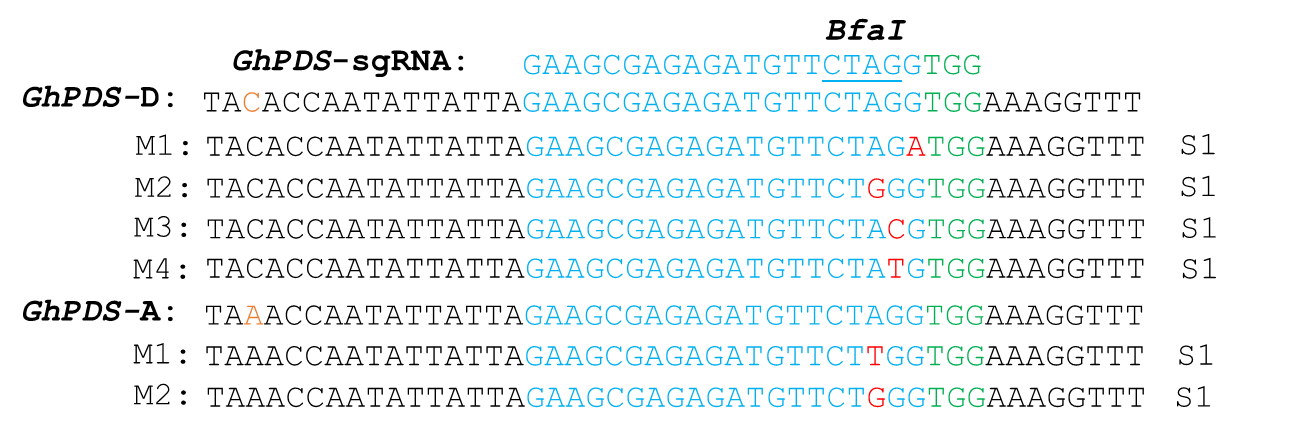
**

**Table 1:** Primer sequences involved in this study

| Primer | Sequence (5'-3') | Application |
| --- | --- | --- |
| Cas9F: | GTCATTACGGACGAGTACAAG | qRT-PCR analysis of *Cas9* mRNA expression |
| Cas9R: | AGGTAGCAGATCCGATTCTTT |  |
| GhUBQ7F: | GAAGGCATTCCACCTGACCAAC | PCR amplification of partial fragments of *GhUBQ7* gene |
| GhUBQ7R: | CTTGACCTTCTTCTTCTTGTGCTTG |  |
| GhMAPKKK2-sgRNAF: | GATTGAGGGTTCCCAGCTGACATA | Construction of sgRNA(*GhMAPKKK2*): 5'-GAGGGTTCCCAGCTGACATA-3' |
| GhMAPKKK2-sgRNAR: | AAACTATGTCAGCTGGGAACCCTC |  |
| GhPDS-sgRNAF: | GATTGAAGCGAGAGATGTTCTAGG | Construction of sgRNA (*GhPDS*): 5'-GAAGCGAGAGATGTTCTAGG-3' |
| GhPDS-sgRNAR: | AAACCCTAGAACATCTCTCGCTTC |  |
| GhCLA1-sgRNA1F: | GATTGGTGATGGTGCTATGACTGC | Construction of sgRNA1 (*GhCLA1*): 5'-GGTGATGGTGCTATGACTGC-3' |
| GhCLA1-sgRNA1R: | AAACGCAGTCATAGCACCATCACC |  |
| GhCLA1-sgRNA2F: | GATTGATGCTCGTGGAATGATCAG | Construction of sgRNA2(*GhCLA1*): 5'-GATGCTCGTGGAATGATCAG-3' |
| GhCLA1-sgRNA2R: | AAACCTGATCATTCCACGAGCATC |  |
| FT-GhPDS-sgRNAF: | CTGAGTTTATATACAGCTAGAGTCGAAGTAGTGATTGATGTCTATAAATATAAGAGACCCTC | Transfer PCR amplification of *FT*-*GhPDS*-sgRNA |
| FT-GhPDS-sgRNAR: | TATTTCTAGCTCTAAAACCCTAGAACATCTCTCGCTTTTGGCCATAAGTAACCTTTAGAG |  |
| FT-GhCLA1-sgRNA1F: | CTGAGTTTATATACAGCTAGAGTCGAAGTAGTGATTGATGTCTATAAATATAAGAGACCCTC | Transfer PCR amplification of *FT*-*GhCLA1*-sgRNA1 |
| FT-GhCLA1-sgRNA1R: | CTCTAAAACGCAGTCATAGCACCATCACTTGGCCATAAGTAACCTTTAGAG |  |
| M-GhPDSF: | GCATAATCACTATCAGAGAACC | Amplification of DNA fragments flanking *GhPDS* target from cotton A, D genome |
| M-GhPDSR: | GATCCATCACTCAAGTTTGTTTTTG |  |
| M-GhCLA1F: | GCAGGCTTGGGTATTAATCC | Amplification of DNA fragments flanking *GhCLA1* target from cotton A, D genome |
| M-GhCLA1R: | GGCTTATTCTGTATGTTACCG |  |
| M-GhMAPPPK2F: | CCATGTCGTAGCTTATAAAGG | Amplification of DNA fragments flanking *GhMAPPPK2* target from cotton A, D genome |
| M-GhMAPPPK2R: | TCATTTACCTTCTCTTCCCAG |  |
| HiTom-GhPDSF: | GGAGTGAGTACGGTGTGCTAATAGAGTAGACTGGCCAC | Detection of *GhPDS* gene editing efficiency by high-throughput sequencing |
| HiTom-GhPDSR: | GAGTTGGATGCTGGATGGGGGGGTAAACTTCTATGAAAC |  |
| HiTom-GhCLA1F: | GGAGTGAGTACGGTGTGCGAAGGGATCTGAAAGGTGAA | Detection of *GhCLA1* gene editing efficiency by high-throughput sequencing |
| HiTom-GhCLA1R: | GAGTTGGATGCTGGATGGCTCTAAGAGGCCTGTTTGAT |  |
| Gh_A08G0193-F: | GGAGTGAGTACGGTGTGCTCAGCTGGTTTAGGTACTTG | Detection of Gh_A08G0193 gene by high-throughput sequencing |
| Gh_A08G0193-R: | GAGTTGGATGCTGGATGGCAAGATGATGATGAGATTAGTGTC |  |
| Gh_A12G0784-F: | GGAGTGAGTACGGTGTGCGAAGAAGGTCCAGGATGCAT | Detection of Gh_A12G0784 gene by high-throughput sequencing |
| Gh_A12G0784-R: | GAGTTGGATGCTGGATGGAACCCTGCATTATTCATAGCC |  |
| Gh_D13G1262-F: | GGAGTGAGTACGGTGTGCATCCCTACTCCAGTTTAACC | Detection of Gh_D13G1262 gene by high-throughput sequencing |
| Gh_D13G1262-R: | GAGTTGGATGCTGGATGGCAAGAACTTCTATTAACCTGGTTTT |  |
| Gh_A10G0595-F: | GGAGTGAGTACGGTGTGCACCCGTTATGCTGGACCGAA | Detection of Gh_A10G0595 gene by high-throughput sequencing |
| Gh_A10G0595-R: | GAGTTGGATGCTGGATGGAGAGAAAAGCGCGACAACAA |  |
| Gh_A10G0237-F: | GGAGTGAGTACGGTGTGCCTTCCGACATTAGCTTATCATC | Detection of Gh_A10G0237 gene by high-throughput sequencing |
| Gh_A10G0237-R: | GAGTTGGATGCTGGATGGGGCAAGATTCATATGAATTTCTACC |  |

**Nucleotides sequences of the 5 detected potential off-target sites**

**> Gh_A08G0193 (234 bp)**

GCTAATTAATGGTGGGCTGTTTTATTGCAGGGATGGCAGTTGGAAGAGATCTGTTAGGGAAGAACAATCATGTAATTGCAGTAATAGGTGATGGAGCAATGACTGCTGGAATGGCATATGAAGCACTCAACAATGTTGGTTATCTTGACACTAATCTCATCATCATCTTG

**> Gh_A12G0784 (238 bp)**

GAAGAAGGTCCAGGATGCATACCATAAGAAAAACTTCAGGCCTTGCAGGGTTTCCTAAAAGGGATGAAAGTGTTTATGATGCTTTTGGTGCTGGACATAGTTCCACTAGCATATCAGCTGGACTCGGTATGGCAGTGGCTAGAGACCTTTTGAAGAAGAAAAACAATGTGATTTCAGTGATTGGAGATGGAGCTATGACTGCTGGACTTGCATATGAGGCTATGAATAATGCAGGGTT

**> Gh_D13G1262 (287 bp)**

ATCCCTACTCCAGTTTAACCATTTCTTAACATTAGCAATAGATAAACTTTGGTTGTGGATTTAGTTGCAGATACGGTTATGGTGTCATGATTGCAGGTGTCATGGATGTTACCTAGCTTGTCGTGGTTGTCGAGTCACGAATGTGATTTATTTTTTCTAAAATTTCCATACATGTATATTGTAATATCTTGTACACATAATGCTTATGTTAATTAGATTCTTTAGTTAACTTGCAATATTAATCGCAAACAAAATAATATCTAAAACCAGGTTAATAGAAGTTCTTG

**>Gh_A10G0595 (256 bp)**

ACCCGTTATGCTGGACCGAACCCTCTCTTCCCGCCGCCCTCAGCCCCACCTCGATTTCGACTTTCCCTCCACCGCCGCATCTTCCGCCGTGTCTCCCGTCCCCGAATCCCCCACCTCACTCGCCGACGAGTCCAAAACCAAGAAGCCACCCCTCTATCTCTTGGCCACCAATTACATTTCTCGCTTCGGTCTCATCAAATCGCCGTGTCTCTGCCTCTCCCTTTGCCTTCTTCTCATTGTTGTCGCGCTTTTCTCT

**>Gh_A10G0237 (257 bp)**

CTTCCGACATTAGCTTATCATCTTTTGTCCAAAACTATTGTATTATCTTCTTTCTGACACTTCCTGGCAGCATATATACCTAAAGCTTACCTTTATTGGTAGAGGTAGAGGGTGATAAATGGAATTTTGGATTAGATTATGTTCTTAGAACTTCTTGATGAAGAGAGATTTGTGCTAGGTGGGTGGCTAAAGTGATATGTCACACCACATAACATGTATACATGAAGGATATGGTAGAAATTCATATGAATCTTGCC

Blue nucleotides indicate the potential off-target sites, red nucleotides indicate the PAM sequence.
